# Predictors of depression, stress, and anxiety among non-tenure track faculty

**DOI:** 10.3389/fpsyg.2014.00701

**Published:** 2014-07-08

**Authors:** Gretchen M. Reevy, Grace Deason

**Affiliations:** ^1^Department of Psychology, California State University, East BayHayward, CA, USA; ^2^Department of Psychology, University of Wisconsin-La CrosseLa Crosse, WI, USA

**Keywords:** contingent faculty, stress, depression, organizational commitment, temporary workers

## Abstract

Nationwide in the United States, 70% of faculty members in higher education are employed off the tenure-track. Nearly all of these non-tenure-track (NTT) appointments share a quality that may produce stress for those who hold them: contingency. Most NTT appointments are contingent on budget, enrollment, or both, and the majority of contingent faculty members are hired for one quarter or semester at a time. Significant research has investigated the effects of contingency on teaching, students, departments, colleges, and universities; however, little research has focused on the psychological experiences of NTT faculty. The current study examined perceptions of workplace stressors and harm, organizational commitment, common coping mechanisms, and depression, anxiety and stress among NTT faculty using a longitudinal design that spanned 2–4 months. Results indicate that NTT faculty perceive unique stressors at work that are related to their contingent positions. Specific demographic characteristics and coping strategies, inability to find a permanent faculty position, and commitment to one's organization predispose NTT faculty to perceive greater harm and more sources of stress in their workplaces. Demographic characteristics, lower income, inability to find a permanent faculty position, disengagement coping mechanisms (e.g., giving up, denial), and organizational commitment were associated with the potential for negative outcomes, particularly depression, anxiety, and stress. Our findings suggest possibilities for institutional intervention. Overall, we argue that universities would be well-served by attending to the needs of NTT faculty on campus in order to mitigate negative outcomes for institutions, students, and faculty.

## Introduction

Over the past four decades, colleges and universities in the US have seen an increase in the number of non-tenure-track (NTT) faculty. Nationwide, 70% of faculty members in higher education are employed off the tenure-track (Curtis and Thornton, 2013). Most NTT appointments are contingent on budget, enrollment, or both, and the majority of NTT faculty are hired for one quarter or semester at a time. Contingent NTT faculty are paid less than assistant professors (Ehrenberg and Zhang, 2005), often do not receive adequate health and retirement benefits (CAW, 2012), are not typically represented in university governance, and academic freedom among such faculty is not protected (Baldwin and Chronister, 2001; AAUP, 2013). For these reasons, the relation of tenure-track and NTT faculty on college campuses has been referred to as a faculty “caste system” (Gappa and Leslie, 1993). In addition to the issues of inequality that arise from differential access to resources and power, NTT faculty are also disproportionately female (Bland et al., 2006; Gappa et al., 2007; Curtis, 2014) and are more likely than tenured or tenure-track faculty to be Black/African American, Hispanic/Latino, or American Indian than to be Asian or White (Bradburn et al., 2002; Curtis, 2014); thus, issues facing NTT faculty also have implications for gender and race equality.

There are a variety of explanations for these trends in higher education. According to some, declines in federal and state education spending, competition from virtual and for-profit colleges, increases in tenured/tenure-track faculty salaries, and greater demand for services to accommodate a diverse student population have forced many institutions to freeze hiring for permanent positions and to hire temporary employees instead (Gappa and Leslie, 1993; Baldwin and Chronister, 2001; Murphy, 2009). However, the American Association of University Professors (AAUP, 2014) claims that the hiring of contingent faculty is not economically necessary, but rather, is the result of a choice to prioritize investment in technology and facilities over investment in instruction. Bok (2006) presents another reason why universities hire NNT faculty: NTT hires may signal the universities' commitment to undergraduate education amid criticism of a research-focused academic culture, thus attracting undergraduate enrollment. There are also important disciplinary differences in the number and nature of NTT faculty on US campuses. In some disciplines (e.g., political science, psychology, philosophy), at Bachelor's degree granting and higher institutions, the majority of NTT faculty hold terminal degrees whereas this may not the case in some other disciplines (e.g., English, math). Moreover, faculty in the liberal arts are more likely to desire full-time work, and to piece together a variety of teaching appointments, whereas business, nursing, and professional faculty are more likely to take on a single course to supplement a full-time job (Lewis, 2012).

Many questions have been raised about the impact of NTT faculty on institutions, tenured/tenure-track faculty, and students, and research in the field of education has begun to address these questions. Although some research examines contingency from the perspective of NTT faculty—their motivations for choosing the work, perceptions of the academic environment, and factors that affect their job satisfaction and organizational commitment—less research has focused on the psychological well-being of NTT faculty. Given the financial pressures that academic institutions have faced since the economic recession of 2008, we believe it is particularly important to examine the psychological impact of the trend toward contingent faculty labor in order to anticipate and mitigate harm to NTT faculty, as well as to institutions and to students.

In an effort to fill this gap, we examine perceptions of workplace stressors and harm, organizational commitment and identification, common coping mechanisms, and the occurrence of depression, anxiety, and stress among NTT faculty using a longitudinal design that spanned 2–4 months. In this research we are examining both “stressors” which are events or conditions which are perceived as stressful, and “stress,” which is the psychological and physiological reaction to a stressor or stressors. We propose that NTT faculty from some demographic groups—specifically, those with lower household incomes and those who desire a tenure-track position—will be more likely to perceive sources of stress (stressors) in the workplace and to report harm to themselves and colleagues in the wake of the 2008 recession. We also expect that commitment to and identification with one's institution and reliance on maladaptive coping mechanisms will predispose NTT faculty to higher levels of depression, anxiety, and stress. Our findings indicate that NTT faculty's experiences of stressors and depression, anxiety, and stress do vary based on demographic characteristics, coping mechanisms, and organizational identification and commitment. More broadly, they suggest a need for further research to examine the broad psychological impact of labor practices in higher education.

The increase in contingent faculty positions has had an impact on universities and students. As noted above, research has begun to examine the effects of contingent faculty hiring on institutions of higher learning. Many see the increase in temporary faculty positions as a threat to academic freedom and tenure, which many professors and universities consider crucial for the development of new knowledge (Chait, 1997; Baldwin and Chronister, 2001; AAUP, 2013). An increase in the number of NTT faculty, and corresponding decrease in the number of tenured faculty, means that there are fewer faculty to participate in shared governance and that more power may shift to administrators (Baldwin and Chronister, 2001; Bradley, 2004; AAUP, 2013). Increased reliance on NTT faculty also creates stratification among faculty, which raises concern over the ability for faculty to build collegial relationships (Thompson, 2003; AAUP, 2013) and the potential for stigmatization or disrespect of NTT faculty (Barker and Christensen, 1998; AAUP, 2013).

Research has also examined the impact of contingent faculty on time spent with students and on faculty activities which are likely to affect student learning. The results generally reveal that the activities of TT and full-time NTT faculty are likely to be more advantageous for student learning than are the activities of part-time NTT faculty. Part-time NTT faculty spend less time interacting with students (Baldwin and Chronister, 2001; Umbach, 2007) and less time preparing for class (Umbach, 2007) than their TT counterparts. Full-time NTT faculty, however, spend more time preparing for class than TT faculty (Umbach, 2007). Part-time contingent faculty are less likely to engage in student-centered and learning-centered classroom activities that have been shown to promote student learning (Umbach, 2007; Baldwin and Wawrzynski, 2011); TT and NTT full-time faculty do not differ in regard to frequency of use of these activities (Umbach, 2007). NTT faculty tend to give higher final grades than TT faculty, possibly in response to pressure to show strong course evaluations in order to secure a future teaching appointment (Sonner, 2000; Johnson, 2011). The negative outcomes which are sometimes associated with part-time NTT positions occur not because part-time NTT faculty don't care about students—to the contrary, evidence suggests that NTT faculty are dedicated, highly qualified teachers (Gappa and Leslie, 1993; Baldwin and Chronister, 2001). Despite their best intentions, however, NTT faculty—particularly those who are part-time—may not have the resources they would need to create a good educational experience for students, such as an on-campus office to meet with students, material resources to facilitate classroom activities, and reasonable course loads that would give them time to grade in-depth exams and assignments (Curtis and Jacobe, 2006; Jaeger et al., 2007).

Previous research has also examined the effects of contingent faculty hiring on the experiences of faculty themselves. One question of interest is, why do faculty choose or accept temporary work? Like many of their TT counterparts, faculty employed off the tenure-track have a strong intrinsic motivation to teach (Dutton, 2009; Lewis, 2012). Academic work successfully satisfies these desires—in general, research shows high job satisfaction among both TT and NTT faculty. However, satisfaction also depends to some extent on demographic and individual difference characteristics, including age and marital status (Schulz, 2009), academic discipline (Palmer, 2002), level of compensation and desire for full time employment (Lewis, 2012), as well as psychological factors like the level of stress associated with the work (Hagedorn, 2000).

Despite the joys of teaching and generally high levels of satisfaction among NTT faculty, temporary academic work can be associated with hardship. As mentioned earlier, NTT faculty are paid less than TT faculty and often receive few or no health or retirement benefits (CAW, 2012). Also, the contingent nature of many NTT positions puts faculty at risk for chronic unemployment or underemployment, and in the US, the unpredictable nature of enrollments makes it extremely difficult for NTT faculty to receive support from unemployment insurance (NFM, 2012). Moreover, many NTT faculty are not permitted to participate in university governance to the level they would prefer (Berret, 2008). Although some faculty do choose contingency, with all of its costs, an estimated 50% (CAW, 2012) use part-time or temporary teaching as a substitute for full-time work, and would prefer a full-time or tenure-track position. Tenure-track positions include many of the benefits of faculty work without the financial risks and stigma of many NTT positions.

Another question of interest is the impact of contingency on organizational commitment and identification among faculty. Organizational commitment is defined as an employee's level of dedication to a specific organization (Meyer and Allen, 1991; Murphy, 2009). According to theories of commitment developed in organizational psychology, commitment develops when individual employees feel valued by an organization (Meyer and Allen, 1991) and is a key determinant of retention and performance (Mowday et al., 1982; Rhoades and Eisenberger, 2002). A related concept, organizational identification, is often used interchangeably with organizational commitment, but is a distinct characteristic defined as the perception of unity with or belonging to an organization (Ashforth and Mael, 1989). Employees who are high in organizational identification define themselves, in part, in terms of the organization, and are likely to experience the group's victories and setbacks as their own (Ashforth and Mael, 1989). Once an individual identifies as a member of the organization, there is potential for commitment to develop.

Given the importance of retaining a dedicated and talented faculty, organizational commitment and identification should be of interest to academic institutions. Organizational commitment, in particular, offers rewards to employees in the form of intrinsic motivation and satisfaction (Mowday et al., 1982; Koys, 1988). Unfortunately, the contingent nature of many NTT academic positions sends an implicit message to NTT faculty that they are not valued by the organization. In doing so, universities may limit the depth of the exchange between employer and employee that is so important for the development of commitment. In line with this, studies of temporary work arrangements in non-academic contexts find that contingent workers are less committed and less effective than their permanent counterparts (Blau, 1964; McGinnis and Morrow, 1990; Pearce, 1993; Kallenberg, 2000; Liden et al., 2003; Connelly and Gallagher, 2004; Kraimer et al., 2005).

Rather than accepting lower commitment of contingent employees as a given, research has aimed to identify antecedents of organizational commitment that can be strengthened to increase levels of dedication among employees. Predictors of organizational commitment identified in the research include recognition, support, compensation, and participation in shared governance (Wayne et al., 1997; Kacmar et al., 1999; Murphy, 2009). Characteristics of individual employees are also associated with organizational commitment. Age and marital status predict organizational commitment such that older employees and those who are married show higher levels of commitment (Kacmar et al., 1999). Women also demonstrate higher levels of organizational commitment than men (Mathieu and Zajac, 1990; McFarlin and Sweeney, 1992).

Given that organizational commitment is a psychological state, it is more than a desirable end goal for faculty and institutions. Organizational commitment is also likely to shape employees' reactions to workplace situations. Although examinations of the moderating role of organizational commitment are not as common in the literature, Groff (2012) found initial evidence that organizational commitment can buffer the effect of a negative workplace event on employee turnover, likely because dedication to the organization motivates committed employees to interpret new workplace developments in more positive ways. In the case of NTT faculty, however, organizational commitment may operate differently than it operates for permanent employees since NTT faculty may be committed to an organization that is not committed to them. Possibly, among NTT faculty organizational commitment may not operate as a buffer of the effects of negative workplace events, but rather, feeling commitment to an organization that fails to reciprocate the commitment may lead to negative reactions such as depression, anxiety, or stress.

The many challenges of contingent work also raise the possibility that NTT faculty experience high levels of stress, anxiety, and depression. The conventional wisdom is that job insecurity that stems from temporary work status can contribute to a variety of health problems, and indeed, several studies conducted in non-academic workplace settings have found that temporary employees are more likely to have health problems (Benavides and Benach, 1999; Benavides et al., 2000). Stress at work is also associated with temporary work status in non-academic settings, such that temporary workers report more stress than permanent employees (Benavides et al., 2000). Higher levels of stress are also associated with lower job satisfaction (Hagedorn, 2000), organizational commitment (Meyer and Allen, 1997), and employee retention (Barnes et al., 1998).

To our knowledge, depression and anxiety have not been studied in the temporary faculty population. Studies comparing the stress levels of part-time and full-time faculty find that part-time faculty experience less stress (Gappa and Leslie, 2002; Outcalt, 2002). This difference may be explained by occupational role overload experienced by full-time faculty, a feature of academic work that full-time NTT faculty are also likely to experience (Barnes et al., 1998; Lease, 1999). However, the nature of stressors that NTT faculty experience may be different and may be high under certain circumstances, such as an economic recession. Stress over the stigma and status of their rank may also be a unique stressor for NTT faculty. Given the possible link between workplace stressors and other important personal and institutional outcomes among NTT faculty, understanding the impact of contingency on stress, depression, and anxiety among this population is an important goal.

The coping mechanisms that NTT faculty use may also affect both their stress levels and other psychological outcomes, including anxiety and depression. The current study uses a widely used measure of coping, the COPE scale (Carver et al., 1989), which assesses a diverse range of 15 coping mechanisms. Numerous researchers have produced models of the structure of coping, which involves categorizing coping mechanisms into a relatively small number of general categories (Connor-Smith and Flachsbart, 2007). One category system involves classifying coping mechanisms as involving either *engagement* with or *disengagement* from the stressor, i.e., either purposefully undertaking to manage the stressor and/or its related emotions, or distancing oneself from the stressor and/or emotions. For instance, the COPE scales “planning” and “active coping” are classified as engagement mechanisms whereas “denial” and “behavioral disengagement” (giving up) are classified as disengagement mechanisms. Although it is not possible to claim that a particular coping mechanism is either functional or dysfunctional in all contexts, roughly speaking, use of engagement coping mechanisms tends to be associated with more positive life outcomes, including better physical and mental health, whereas use of disengagement coping mechanisms tends to produce less desirable outcomes (Compas et al., 2001).

Research on the impact of contingent faculty in higher education has tended to focus on the impact of contingency on universities and on students, with less attention paid to the experiences and well-being of faculty. As described above, previous literature leaves several unanswered questions about contingent faculty experiences of stress and the impact of contingency on longer-term health outcomes such as depression and anxiety. The psychological literature on stress and coping suggests new ways of understanding the experience of contingency, and points to questions that are important for the future of faculty well-being:

(1) What are the key workplace stressors for contingent faculty?

This is a particularly important question in the wake of the 2008 recession, which continues to impose a financial strain on many universities. NTT faculty may report that their primary stressors are those that are related to financial security (e.g., contingency, low pay, no health insurance). The distinct nature of faculty work and the high levels of job satisfaction among NTT faculty may further bolster the idea that when NTT faculty identify primary workplace stressors, the stressors would tend to be unrelated to their actual work tasks (e.g., lecturing, interacting with students), since the work itself is experienced as rewarding.

(2) How do NTT faculty cope with stress related to contingency? To what extent do they use adaptive or maladaptive coping strategies, and what are the broader health consequences of these strategies?

Previous research links temporary employment to negative health outcomes. Again, because this research is conducted in non-academic settings, it is unclear whether contingent employment in academia is a risk factor for health. The psychological literature on stress and coping indicates that not only stress level, but also the method for coping with stress can put employees at risk for negative health outcomes. In particular, disengagement coping mechanisms such as behavioral disengagement (giving up) and denial are likely to be linked to negative health outcomes such as anxiety and depression (Allman et al., 2009; Gandy et al., 2012; Li et al., 2014).

(3) What are sources of individual variation in contingent faculty experiences of stress?

Previous research indicates that contingent faculty may experience the workplace differently based on demographic factors. Gender influences perceptions of stressors, in both work and non-work environments. Results of a large meta-analysis reveal that women report more stressors than men across many life domains (Davis et al., 1999). Age is also related to perceived stressors. Older people report significantly lower levels of daily hassles stressors (minor stressors), such as getting stuck in traffic or running late for an appointment, than do younger people (Aldwin, 2011). It is not clear whether older people experience fewer of these types of stressors or whether they are simply less bothered by such potentially annoying events. Aldwin (2011) states that the latter interpretation may be most likely. Age may not be related to absolute amount of major stressors, such as death of a loved one or divorce; research results are inconsistent (Aldwin, 2011).

In addition, the psychological literature on stress and coping indicates systematic variation in how individuals cope with stress. In a meta-analysis on sex comparisons in coping, Tamres et al. (2002) report that women engage in more positive self-talk (encouraging oneself), seeking social support, and rumination than do men. Coping may also change with age, such that older adults may utilize more efficient coping mechanisms, those which conserve resources, appear to be better at regulating negative emotions, and may be less likely to appraise situations as highly stressful (Aldwin, 2011). Therefore, we might expect that NTT experiences of stress and coping will vary based on these individual differences.

The current study involves investigating perceived workplace stressors, perceived workplace harm, coping, organizational commitment and identification, and well-being outcomes related to these variables (depression, anxiety, and stress) among contingent faculty members. A first goal is to describe common stressors and coping mechanisms for contingent faculty. We will also examine the degree of organizational commitment among contingent faculty, and investigate whether commitment correlates with age and gender, as shown in earlier research. Next, we will predict perceptions of workplace stressors and harm, depression, anxiety, and stress from general demographic variables, situational variables (i.e., total family income and desire for permanent faculty work), and individual coping mechanisms, with the general expectation that variables at each of these psychological levels will partially explain the psychological experiences of NTT faculty. In predicting depression, anxiety, and stress, which were measured at time 2, we will utilize measures of harm, stressors, coping mechanisms, organizational commitment and organizational identification which were taken at time 1, in order to determine if these presumably stable personal qualities (i.e., coping mechanisms, identification, and commitment) and perceived situational factors (i.e., perceived stressors and harm), which may or may not be stable, precede the psychological reactions of depression, anxiety, and stress. Lastly, we will investigate the interaction between organizational commitment and stressors/harm as predictors of depression, anxiety, and stress. This last set of analyses will address the question: Does commitment buffer the effects of stressors and harm on depression, anxiety, and stress, as might be predicted from research on other types of workers, or will we find that among contingent faculty, organizational commitment fails to operate as a buffer? We predict the latter result, given that organizational commitment may operate as a protective factor only among those employees who feel job security in their positions.

## Materials and methods

### Procedure

The procedures of this study were approved by the Institutional Review Board of California State University, East Bay. We solicited participants for an online two-part study on experiences of contingent faculty between October 2011 and April 2012. Solicitations were sent to four listservs: adj-l (which focuses on adjunct faculty issues), the Society for Personality and Social Psychology, the Society for the Psychological Study of Social Issues, and the Society for the Teaching of Psychology. All lecturers at a medium-sized public university in the United States were invited to participate. Additionally, the New Faculty Majority, an advocacy organization for contingent faculty, posted the first solicitation on their website. Prospective participants were told that, for the purposes of this survey, contingent faculty members were defined as any instructional or research faculty who work off the tenure track at institutions of higher education, such as lecturers, adjunct faculty, post-docs, and graduate students.

The survey was through Google docs. Once at the Google docs website, participants gave their informed consent. They provided an email so that we may send them a reminder email about participating in Part 2, and were asked to create a unique ID number so that we could match Part 1 and Part 2 surveys. Next, participants completed a series of questionnaire measures in the following order: organizational commitment, organizational identification, perceptions of stressors, perceptions of harm, coping, and a demographic information form. Immediately prior to the questionnaire about perception of stressors, participants were asked an open-ended question: Which aspects of your job do you find most stressful? If possible, list and describe at least two aspects. Additionally, participants were invited to make comments about the survey or anything else. Lastly, participants were thanked, given contact information of the researchers, and were invited to include their name, email address, and phone number if they were interested in participating in an optional drawing for one of five $100 gift cards redeemable at Powells Books, an independent bookseller headquartered in Portland Oregon, which sells books both onsite and online.

Two to four months after participation in the Part 1 survey, between January and July of 2012, participants received an email inviting them to complete the Part 2 survey. Participants completed the following questionnaire measures: perceptions of stressors, perceptions of harm, coping, depression, anxiety, and stress, organizational commitment, and organizational identification. Next, the participants were thanked, provided with the researchers' contact information, and were invited to enter their name, email address, and phone number if they would like to participate in the drawing. Lastly, the participants were brought to a debriefing page that explained that the study involved examining relationships between stress and coping strategies and contingent faculty health outcomes and workplace commitment.

### Participants

#### Part 1 survey

There were 199 participants (129 women, 67 men, and three who did not report a gender) in the Part 1 survey. Demographic characteristics which are expressed as percentages of the sample (e.g., racial/ethnic background, highest education level) are presented in Table 1. Age of participants ranged from 24 to 85 with a mean of 47.9. Family incomes ranged from under $10,000 per year to over $150,000 per year with the mean income in the $50,000–59,000 range. Among those who are married, most (59.8%) reported that their spouse worked 40 or more hours per week in paid employment. Paid hours of work per week varied widely, ranging from 2 to 60, with many reporting that they did not know the answer. Average number of years working in higher education in a temporary teaching and/or research position was reported as 10.7 (range from 1 to 50), with 87.4% of the sample responding. In response to the question, “How many years have you been in your current job that you consider your primary job?” number of years ranged from 0.25 to 44, with a mean of eight. Five percent did not respond to the question, 2.5% were retired, and 1% were unemployed. In response to the question, “At how many institutions of higher education do you typically work during any one “term” (semester or quarter)”? the mean was 1.50 and the number ranged from one to five. Nine percent of people did not respond to the question.

**Table 1:** **Participants' demographic characteristics for Part 1 of the survey**.

**Demographic variable**	**% in survey sample**
**RACIAL/ETHNIC BACKGROUND**	
Asian/Asian American	2.0
Black/African American	3.5
Latino/Hispanic	3.0
Native American	0.5
White/Caucasian	81.4
Multiracial	3.0
Other	1.0
Missing	5.5
**CITIZENSHIP**	
United States	91.5
Other country	6.0
Missing	2.5
**HIGHEST EDUCATION LEVEL**	
Doctorate	40.7
Juris doctor	1.0
ABD (all but dissertation)	15.1
Master's degree	38.7
Bachelor's degree	3.5
Missing	1.0
**FIELD IN WHICH HIGHEST DEGREE WAS EARNED**	
Art, film, theater, or music	7.5
Anthropology	3.5
Biology	2.0
Business, economics, or management	3.0
Education	7.0
English, literature, creative writing, or language	20.5
History	6.5
Psychology, counseling, or social work	18.5
Other	27.5
Missing	4.0
**MARITAL/RELATIONSHIP STATUS**	
Married	47.7
Unmarried and cohabiting	8.0
Divorced	11.1
Single, never married	26.1
Separated	1.0
Widowed	4.0
Missing	2.0
**CHILDREN**	
Yes	48.2
No	48.7
Missing	3.0
**TYPE OF CONTINGENT POSITION**	
Adjunct/contingent faculty or instructor	84.9
Teaching assistant or graduate student instructor	6.5
Research associate or post-doc	4.0
Other	2.0
Missing	2.5
**LOCATION OF PRIMARY JOB**	
University that grants graduate degrees	50.8
Four-year college	16.6
Community college	23.1
Missing	9.5
**RECEIVE BENEFITS THROUGH OWN EMPLOYMENT, SPOUSES' EMPLOYMENT, OR OTHER SOURCE?**	
No	29.1
Health insurance only	18.1
Retirement only	8.0
Both health insurance and retirement	38.2
Health, retirement, and other benefit	2.0
Don't know or sometimes	2.0
Missing	2.0

#### Part 2 survey

Ninety participants (54 women, 35 men, and one who did not identify a gender) participated in both the Part 1 and Part 2 surveys. Demographic characteristics which are expressed as percentages of the sample (e.g., racial/ethnic background, highest education level) are presented in Table 2. Age ranged from 25 to 79 with a mean of 49.4. Family incomes ranged from $10,000 to over $150,000 per year, with the mean income in the range of $60,000–69,999. Among those who are married, most (68.8%) reported that their spouse worked 40 or more hours per week in paid employment. Paid hours of work per week varied widely, ranging from 2 to 60, with many reporting that they did not know the answer. Average number of years working in higher education in a temporary teaching and/or research position was reported as 12, with 85.5% of the sample responding. In response to the question, “How many years have you been in your current job that you consider your primary job?” number of years ranged from 0.25 to 44, with a mean of 8.3. Two percent did not respond, 3.3% were retired, 1.1% was unemployed. In response to the question, “At how many institutions of higher education do you typically work during any one “term” (semester or quarter)”? the mean was 1.53 and the number ranged from one to five.

**Table 2:** **Participants' demographic characteristics for Part 2 of the survey**.

**Demographic variable**	**% in survey sample**
**RACIAL/ETHNIC BACKGROUND**	
Black/African American	3.3
White/Caucasian	85.6
Multiracial	3.3
Other or missing	7.7
**CITIZENSHIP**	
United States	94.4
Other country	4.4
Missing	1.1
**HIGHEST EDUCATION LEVEL**	
Doctorate	46.6
ABD (all but dissertation)	18.9
Master's degree	31.1
Bachelor's degree	3.3
**FIELD IN WHICH HIGHEST DEGREE WAS EARNED**	
Art, film, theater or music	6.6
Anthropology	5.5
Biology	3.3
Business, economics, or management	4.4
Education	9.9
English, literature, creative writing, or language	14.4
History	6.7
Nursing or health sciences	3.3
Philosophy	3.3
Psychology or counseling	21.0
Other	20.9
**MARITAL/RELATIONSHIP STATUS**	
Married	52.2
Unmarried and cohabiting	5.6
Divorced	13.3
Single, never married	23.3
Separated	1.1
Widowed	3.3
Missing	1.1
**CHILDREN**	
Yes	47.8
No	50.0
Missing	2.2
**TYPE OF CONTINGENT POSITION**	
Adjunct/contingent faculty or instructor	86.7
Teaching assistant or graduate student instructor	5.6
Research associate or post-doc	4.4
Other	3.3
**LOCATION OF PRIMARY JOB**	
University that grants graduate degrees	56.7
Four-year college	15.6
Community college	20.0
Missing	7.8
**RECEIVE BENEFITS THROUGH OWN EMPLOYMENT, SPOUSES' EMPLOYMENT, OR OTHER SOURCE?**	
No	32.2
Health insurance only	18.9
Retirement only	6.6
Both health insurance and retirement	40.0
Health, retirement, and other benefit	2.2

### Measures

The Affective Commitment Scale (ACS; Allen and Meyer, 1990) was used to measure organizational commitment to a university where one works, in both the Part 1 and Part 2 surveys. The instructions regarding which university to choose were, “Please respond to the following items about the college or university where you work. If you work at more than one institution, respond to the items in regard to the institution about which you feel most positively.” The ACS consists of eight items, each of which is rated from 1 (strongly agree) to 7 (strongly disagree). Total scale scores range from 7 to 56. The eight items were modified to replace the word “organization” with “college/university.” An example of an item is: “I would be very happy to spend the rest of my career with the college or university where I work now.” Half of the items are reverse scored. Allen and Meyer (1990) report an alpha of 0.87 for the ACS. As validity evidence they report that the ACS correlates significantly with the Organizational Commitment Questionnaire (Mowday et al., 1979). Alphas in the current study were in the 0.80 s for the Part 1 and Part 2 surveys and for the combined data set.

The Organizational Identification Questionnaire (OIQ; Mael and Ashforth, 1992) was used to measure organizational identification with a university where one is employed, in both the Part1 and Part 2 surveys. The instructions regarding which university to choose were, “Please respond to the following items about the college or university where you work. If you work at more than one institution, respond to the items in regard to the institution about which you feel most positively.” The OIQ consists of six items, each of which was rated from 1 (strongly agree) to 7 (strongly disagree) in the current study. Total scale scores range from 6 to 42. The six items were modified to use the expression “college/university” or a relevant variant. An example of an item is “This college/university's successes are my successes.” Mael and Ashforth (1992) report coefficient alphas in earlier studies, all in the 0.80 s. Alphas in the current study were in the 0.80 s for the Part 1 and Part 2 surveys and for the combined data set.

The researchers developed a questionnaire to measure stressors, called “Contingent Faculty Stressors Questionnaire (CFSQ),” which was used in both the Part 1 and Part 2 surveys. The CFSQ consists of five items which ask the following: whether overall stress level has changed since the economic downturn of 2008, whether job security has decreased, whether income has decreased, whether workload has increased, and whether medical benefits were lost at some point since 2008. The Part 2 survey asked respondents to rate which of the same aspects of their work have changed since their completed the Part 1 survey and to rate the degree of change. Higher scores indicated more stress (e.g., overall stress level changing for the worse, job security decreasing, etc.). All variables ran from 0 to 1 such that total scores on the stressor scale ranged from 0 to 5. Alphas in the current study were 0.67 for the time 1 survey, 0.67 for the time 2 survey, and 0.70 for the combined data set. A factor analysis, using a Maximum Likelihood Analysis with direct oblimin rotation revealed that all stressor items loaded on a single factor.

The researchers developed a questionnaire to measure perceived harm observed in the workplace, called “Contingent Faculty Harm Scale (CFHS),” which was used in both the Part 1 and Part 2 surveys. The CFHS consists of four items which ask whether the participant has observed the following: harm occurring to colleagues, for instance, loss of job or loss of income; harm occurring to students, for instance, increased tuition or decreased access to classes; people at work treating one another more poorly than they used to; and personally having less freedom to speak his/her mind in the classroom and/or with colleagues and administrators. The Part 2 survey asked respondents to rate which of the same aspects of their work have changed since their completed the Part 1 survey and to rate the degree of change. Each of the four items is rated on a four-point scale where 0 = not at all, 1 = somewhat, 2 = moderately, and 3 = dramatically. Scores on the Harm questionnaire range from 0 to 12. Alphas in the current study were 0.69 for the time 1 survey, 0.74 for the time 2 survey, and 0.72 for the combined data set. A factor analysis using a Maximum Likelihood Analysis with direct oblimin rotation revealed that all harm items loaded on a single factor.

The COPE scale (Carver et al., 1989) was used to measure coping mechanisms in both the Part 1 and Part 2 surveys. The COPE scale measures 15 coping mechanisms: active coping, planning, suppression of competing activities (putting aside other activities and concerns in order to focus on the stressor), restraint coping (waiting till the right time), seeking instrumental support, seeking emotional support, positive reinterpretation and growth, acceptance, turning to religion, venting, denial, behavioral disengagement (giving up), mental disengagement, alcohol and drug disengagement (also called “substance use”), and use of humor. The COPE scale includes 60 items; each scale is assessed by four items. Examples of items include “I get upset and let my emotions out” and “I make a plan of action.” Items are rated on a four-point scale: 1 = I usually don't do this at all, 2 = I usually do this a little bit, 3 = I usually do this a medium amount, and 4 = I usually do this a lot. Scores on each scale range from 4 to 16. The COPE scale possesses acceptable reliability and validity; Carver et al. (1989), utilizing a sample of 978 reported alphas ranging from the 0.60 to 0.90 s for all of the scales except mental disengagement, with an alpha of 0.45. In the current study, alphas ranged in the 0.70 to the 0.90 s for all scales for both Part 1 and Part 2 surveys, and for the combined data set, with the exception of mental disengagement, denial, and suppression, for which alphas ranged in the 0.60 s for Part 1 and Part 2 data surveys and for the combined data set.

The Depression, Anxiety, and Stress Scale (DASS; Lovibond and Lovibond, 1995) was used to measure depression, anxiety, and stress in the Part 2 survey. The DASS consists of 42 items, each of which is rated on a four-point scale: 0 = did not apply to me at all, 1 = applied to me to some degree, or some of the time, 2 = applied to me to a considerable degree, or a good part of time, and 3 = applied to me very much, or most of the time. Each of the three scales (Depression, Anxiety, and Stress) consists of 14 items. Scores on each of the three scales range from 0 to 42. Participants were asked to rate how much each of the items applied to them over the past week at work. Examples of items are: “I couldn't seem to experience any positive feeling at all” (Depression), “I experienced trembling (e.g., in the hands)” (Anxiety), and “I found myself getting upset rather easily” (Stress). Lovibond and Lovibond (1995) report alpha coefficients for the three scales ranging from 0.81 to 0.91. Refer to Lovibond and Lovibond (1995) for validity data. In the current study, alphas of the three scales ranged from 0.94 to 0.96.

Desire for a permanent (tenure-track) position was measured with the following item: “Do you accept temporary work due to difficulty finding a permanent position?” This item was rated on a scale from 1 (no, not at all) to 5 (yes, to a large degree). This item was created as part of a five-item survey assessing reasons why faculty choose temporary work and was not originally written to specifically assess whether temporary faculty desire permanent positions; this explains why the item appears to be an indirect assessment of desire for a permanent position. As we began data analysis we determined that this item could be used to assess desire for a permanent position. The other four items of the survey were not utilized in the current study.

### Coding of qualitative data

In the Part 1 survey, participants were asked to answer the following open-ended question: Which aspects of your job do you find most stressful? If possible, list and describe at least two aspects. To capture the content of these responses, one author and a graduate student created a coding taxonomy to classify participant responses into various categories. The coding taxonomy was created based on an initial reading of approximately 20% of the participant responses. The final coding taxonomy comprises 13 distinct categories: contingency/precariousness; lack of respect; not allowed to participate in service/governance and/or department politics; grading; workload; lack of secretarial/colleague/university support (including physical work space); lack of recognition/invisibility; low pay/pay inequity; no benefits (health, etc.); difficult/demanding students; students academically unprepared/disengaged; pressure to write, publish, or conduct research; and pressure to produce high students outcomes/perform at high standards. An “uncodeable/junk” category allowed us to document content of the responses that was not relevant to teaching or university work. An “other” category allowed us to document any additional content related to teaching and university work that was not included in the coding taxonomy categories.

A graduate student coder, blind to study hypotheses, divided each of the participant responses into phrases, clauses, and sentences that expressed a distinct thought or idea; these constituted the text units for coding. Next, she coded each text unit for the presence of the themes captured by our coding taxonomy. The specific instructions given to the graduate student coder were: “Look at the full sentence. If everything in the sentence is junk, code as junk. If parts are not junk, code each distinct idea separately, and do *not* code the rest of the sentence as junk. Any single idea can receive more than one code.” For example, the following response “Total lack of job security, living wage, health benefits, or access to opportunity for real professional development (i.e., being considered for full-time teaching opportunities—I've applied several times and not even been able to get an interview)” was coded as continency/precariousness, low pay/pay inequity, no benefits (health, etc.) and “other” for the reference to professional development, since only a small number of respondents mentioned lack of access to professional development.

Measures created from these codes reflect the proportion of the participants who mentioned a given theme in their response.

### Data analysis

Quantitative data analysis involved the following: We explored relationships between all study variables through Pearson correlations. Also, we conducted five standard multiple regressions, predicting the following variables: perceived harm, perceived stressors, depression, anxiety, and stress. Additionally, we conducted six multiple regressions to examine whether organizational commitment interacts with (a) perceived workplace harm and (b) perceived workplace stress in the prediction of depression, anxiety, and stress.

## Results

All quantitative data were analyzed using SPSS version 21. To correct for potential type I error due to the large number of analyses, alpha levels were set at 0.01.

### Descriptive statistics

Means and standard deviations for organizational commitment, organizational identification, perceived stressors, perceived harm, and COPE scale variables for the Part 1 data set are presented in Table 3. Means and standard deviations for commitment, identification, stressors, harm, and COPE scale variables for the combined Part 1 and Part 2 data sets and DASS variables for the Part 2 data set are presented in Table 4.

**Table 3:** **Means and standard deviations for commitment, identification, perceived stressors and harm, and COPE variables for the Part 1 survey**.

**Variable**	**Mean**	**Standard Deviation**	**Range**
Commitment	31.63	10.03	10–53
Identification	22.89	7.70	6–41
Perceived stressors	2.42	1.23	0.6–5
Perceived harm	5.34	2.96	0–12
Growth	11.12	2.73	4–16
Mental disengagement	8.78	2.68	4–16
Venting	9.60	3.03	4–16
Instrumental support	10.96	3.06	4–16
Active coping	11.95	2.86	4–16
Denial	4.78	1.46	4–11
Religious coping	6.49	3.81	4–16
Humor	9.38	3.43	4–16
Behavioral disengagement	6.18	2.32	4–15
Restraint	10.07	2.72	4–16
Emotional support	11.12	3.47	4–16
Substance use	5.42	2.73	4–16
Acceptance	10.73	2.78	4–16
Suppression	9.35	2.47	4–16
Planning	12.83	2.77	4–16

**Table 4:** **Means and standard deviations for commitment, identification, perceived stressors and harm, COPE and DASS variables for the Part 2 survey**.

**Variable**	**Mean**	**Standard Deviation**	**Range**
Commitment	31.01	9.68	13–53
Identification	22.22	7.58	9–41
Perceived stressors	2.31	1.21	0.6–5
Perceived harm	5.24	3.13	0–12
Growth	10.90	2.68	6–16
Mental disengagement	8.43	2.68	4–16
Venting	9.70	3.05	4–16
Instrumental support	10.86	3.02	4–16
Active coping	11.92	2.67	5–16
Denial	4.70	1.43	4–11
Religious coping	6.13	3.59	4–16
Humor	9.43	3.42	4–16
Behavioral disengagement	6.00	2.25	4–15
Restraint	9.79	2.85	4–16
Emotional support	11.24	3.34	4–16
Substance use	5.17	2.37	4–16
Acceptance	10.76	2.70	4–16
Suppression	9.47	2.46	4–16
Planning	12.70	2.67	6–16
Depression	7.52	9.75	0–41
Anxiety	4.15	7.10	0–38
Stress	10.06	9.97	0–38

### Pearson correlations and test–retest reliabilities

The correlation matrix for age, sex, family income, participants' desire for a tenure-track position, commitment, identification, perceived stressors, perceived harm, and COPE variables for the Part 1 data set is presented in Table 5. We are reporting correlations between variables from the Part 1 data set rather than from the combined data set because demographic variables were measured only in the Part 1 data set. Additionally, the sample size for the Part 1 data set is significantly larger than the sample size for the combined data set (*n* = 199 vs. *n* = 90). The majority of variables that were measured in both the Part 1 and Part 2 surveys showed moderately high to high test–retest reliabilities in the combined data set. The variables which appeared in both surveys were commitment, identification, perceived stressors, perceived harm, and the COPE scales. Commitment and identification had test–retest reliabilities in the 0.80 s. The test–retest reliabilities for all COPE variables, with the exception of four were in the 0.60 and 0.70 s. The four exceptions were acceptance and suppression (0.50 s), religious coping (0.96), and denial (0.08). The test–retest reliability for perceived harm was 0.66 and for perceived stressors was 0.48. The low test–retest reliability for denial may be largely explained by the low variability for that variable (*SD* = 1.43), by far the lowest variability among the COPE variables.

**Table 5:** **Pearson correlations between demographic variables, commitment, identification, perceived harm, perceived stressors, and COPE variables**.

	**1**	**2**	**3**	**4**	**5**	**6**	**7**	**8**	**9**	**10**	**11**	**12**	**13**	**14**	**15**	**16**	**17**	**18**	**19**	**20**	**21**	**22**
1. Age	1.0																					
2. Sex	−0.3	1.0																				
3. Income	0.27^***^	−0.08	1.0																			
4. Perm position	−0.02	0.02	−0.18	1.0																		
5. Identification	0.01	0.09	−0.15	0.21^**^	1.0																	
6. Commitment	0.07	0.04	−0.09	0.33^***^	0.65^***^	1.0																
7. Harm	0.23^***^	0.10	−0.09	0.34^***^	0.17^*^	0.35^***^	1.0															
8. Stressors	0.00	0.23^***^	−0.16	0.38^***^	0.21^**^	0.37^***^	0.52^***^	1.0														
9. Growth	−0.04	0.15	−0.14	−0.03	−0.15	−0.19^**^	0.04	0.02	1.0													
10. Ment disengage	−0.22^**^	0.10	−0.26^***^	0.17	0.11	0.20^**^	0.16	0.16	0.08	1.0												
11. Venting	−0.01	0.14	−0.14	0.21^**^	0.10	0.14	0.21^**^	0.06	0.16	0.39^***^	1.0											
12. Instru support	−0.05	0.17	−0.17	0.06	−0.15	−0.12	0.14	0.00	0.48^***^	0.16	0.47^***^	1.0										
13. Active coping	0.07	0.12	−0.03	0.12	−0.15	−0.18^*^	0.13	0.04	0.52^***^	−0.06	0.29^***^	0.58^***^	1.0									
14. Denial	0.03	0.08	0.02	0.00	0.01	0.11	0.21^**^	0.19^**^	0.00	0.28^***^	0.27^***^	0.05	0.02	1.0								
15. Relig coping	−0.04	0.06	0.04	0.01	−0.12	−0.05	0.13	0.22^**^	0.23^**^	0.09	0.21^**^	0.13	0.15^*^	0.15^*^	1.0							
16. Humor	−0.04	−0.07	−0.13	0.10	0.08	0.02	0.15	0.08	0.20^**^	0.26^***^	0.11	0.18	0.13	0.11	−0.08	1.0						
17. Beh disengage	0.02	0.03	−0.11	0.16	0.29^***^	0.38^***^	0.26^***^	0.19^**^	−0.33^***^	0.39^***^	0.12	−0.13	−0.36^***^	0.34^***^	−0.13	0.03	1.0					
18. Restraint coping	0.15	0.12	−0.12	0.09	0.11	0.09	0.14	0.12	0.24^***^	0.13	0.02	0.18	0.14	0.14	0.19^**^	0.20^**^	0.28^***^	1.0				
19. Emot support	−0.04	0.30^***^	−0.07	0.04	0.02	−0.05	0.13	−0.05	0.38^***^	0.21^**^	0.62^***^	0.70^***^	0.35^***^	0.06	0.12	0.17	−0.12	0.08	1.0			
20. Substance use	−0.26^***^	−0.04	−0.12	−0.01	−0.04	−0.09	0.06	−0.04	0.01	0.38^***^	0.15	0.15	−0.06	0.17	−0.14	0.16	0.20^**^	−0.09	0.14	1.0		
21. Acceptance	−0.13	0.04	−0.18^**^	0.09	0.04	0.02	0.00	0.06	0.28^***^	0.16	−0.02	0.15	0.13	−0.05	−0.02	0.30^***^	0.03	0.34^***^	0.11	0.03	1.0	
22. Suppression	0.17	0.15	0.02	0.10	−0.14	−0.06	0.17	0.17	0.38^***^	0.11	0.39^***^	0.44^***^	0.61^***^	0.16	0.18	0.07	−0.08	0.24^***^	0.30^***^	−0.02	0.06	1.0
23. Planning	0.11	0.22^**^	−0.01	0.09	0.14	−0.18	0.15	0.11	0.54^***^	−0.04	0.17	0.50^***^	0.78^***^	0.01	0.13	0.11	−0.30^***^	0.24^***^	0.34^***^	−0.02	0.12	0.55^***^

Perm Position = desire for a permanent position, Ment Disengage = mental disengagement, Instru Support = instrumental support, Relig Coping = religious coping, Beh Disengage = behavioral disengagement, Emot support = emotional support. Coding of sex: 1 = male and 2 = female.

** p <.01,

*** p < 0.001.

The correlations between DASS variables measured in the Part 2 survey and age, sex, family income, participants' desire for a tenure-track position, commitment, identification, perceived stressors, perceived harm, and COPE variables measured in the combined data set are presented in Table 6.

**Table 6:** **Correlations of DASS variables with demographic variables, commitment, identification, stressors, harm, and COPE variables**.

**Variable**	**Depression**	**Anxiety**	**Stress**
Age	−0.18	−0.11	−0.18
Sex	−0.09	−0.08	−0.17
Income	−0.38^***^	−0.29^**^	−0.30^**^
Permanent position	0.34^**^	0.28	0.39^***^
Commitment	0.32^**^	0.32^**^	0.32^**^
Identification	0.21	0.35^***^	0.20
Perceived harm	0.38^***^	0.39^***^	0.39^***^
Perceived stressors	0.34^***^	0.28^**^	0.30^**^
Growth	−0.16	0.03	−0.02
Mental disengagement	0.32^**^	0.20	0.26
Venting	0.28^**^	0.22	0.39^***^
Instrumental support	0.01	0.12	0.10
Active coping	−0.12	−0.03	0.02
Denial	0.11	0.15	0.21
Religious coping	−0.12	−0.02	0.07
Humor	0.07	0.27	0.18
Behavioral disengagement	0.41^***^	0.23	0.32^**^
Restraint coping	0.03	0.16	0.03
Emotional support	−0.01	0.13	0.09
Substance use	0.39^***^	0.44^***^	0.31^**^
Acceptance	0.00	−0.14	−0.04
Suppression	0.15	0.11	0.20
Planning	−0.23	−0.11	−0.17

Permanent Position = Desire for a permanent position. Coding of sex: 1 = male and 2 = female.

** p < 0.01, two-tailed,

*** p < 0.001, two tailed.

### Perceptions of stressors and harm among contingent faculty

To examine the nature of stressors among contingent faculty in our sample, we looked first to the open-ended questions (qualitative data) in the Part 1 Survey, which asked participants to describe the most stressful aspects of their job. The responses mentioned by 10% or more of the sample were workload (31.9% of the sample), contingency/precariousness of status (31.4%), lack of support (including physical space; 30.4%), low pay or pay inequity (26.5%), not being allowed to participate in service/governance/department politics (18.6%), lack of recognition/invisibility (15.7%), and no benefits (health, etc.; 11.3%).

Next, we examined predictors of perceptions of harm and perceptions of workplace stressors. Perceptions of harm in the work environment correlated significantly and positively with age (*r* = 0.23, *p* < 0.01), desire for a tenure-track position (*r* = 0.34, *p* < 0.001), commitment (*r* = 0.35, *p* < 0.001), perceived stressors (*r* = 0.52, *p* < 0.001), venting (*r* = 0.21, *p* < 0.01), denial (*r* = 0.21, *p* < 0.01), and behavioral disengagement (*r* = 0.26, *p* < 0.001). Perceptions of workplace stressors correlated significantly and positively with sex (*r* = 0.23, *p* < 0.001; women reported higher workplace stressors), desire for a tenure-track position (*r* = 0.38, *p* < 0.001), identification (*r* = 0.21, *p* < 0.01), commitment (*r* = 0.37, *p* < 0.001), harm (*r* = 0.52, *p* < 0.001), denial (*r* = 0.19, *p* < 0.01), religious coping (*r* = 0.22, *p* < 0.01), and behavioral disengagement (*r* = 0.19, *p* < 0.01). The results indicate that desire for a tenure track position, both identification and commitment, and several disengagement coping mechanisms are correlated with perceptions of both harm and stressors in the workplace.

Next, we conducted two multiple regression analyses to determine which variables best predict perceived stressors and perceived harm. Each regression equation included the following variables as predictors: all demographic variables, identification, commitment, either perceived harm or perceived stressors (the variable which was not the dependent variable for the particular equation), and coping mechanisms for which the Pearson correlations with the dependent variable were significant. In the regression model predicting perceived workplace harm, age (β = 0.228, *p* = 0.002) and perceived stressors (β = 0.337, *p* < 0.001) were significant [*F*_(10, 146)_ = 7.56, *p* < 0.001; *R*^2^ = 0.36, adjusted *R*^2^ = 0.31]. In the regression model predicting perceived workplace stressors, sex (β = 0.191, *p* = 0.006, with women reporting higher workplace stressors than men), desire for a tenure-track position (β = 0.223, *p* = 0.003), perceived harm (β = 0.261, *p* = 0.001) and religious coping (β = 0.246, *p* = 0.001) were significant [*F*_(10, 146)_ = 8.79, *p* < 0.001; *R*^2^ = 0.39, adjusted *R*^2^ = 0.35]. Refer to Tables 7, 8 for summaries of these regression results.

**Table 7:** **Standard multiple regression analysis predicting perceived harm in the workplace**.

**Variable**	**β**	**Unstd B**	**Std Error**	**95% CI**
Age	0.23^**^	0.05	0.02	[0.02, 0.08]
Sex	0.04	0.24	0.45	[−0.66, 1.13]
Income	−0.06	−0.05	0.06	[−0.18, 0.07]
Desire perm position	0.13	0.26	0.15	[−0.04, 0.56]
Identification	−0.14	−0.05	0.03	[−0.12, 0.02]
Commitment	0.15	0.05	0.04	[−0.01, 0.10]
Perceived stressors	0.34^**^	0.79	0.20	[0.40, 1.18]
Behavioral disengagement	0.10	0.12	0.10	[−0.08, 0.31]
Denial	0.10	0.20	0.16	[−0.12, 0.51]
Venting	0.10	0.10	0.07	[−0.05, 0.24]

Coding of sex: 1 = male and 2 = female.

** p < 0.01 two-tailed.

**Table 8:** **standard multiple regression predicting perceived stressors in the workplace**.

**Variable**	**β**	**Unstd B**	**Std Error**	**95% CI**
Age	−0.07	−0.01	0.01	[−0.02, 0.01]
Sex	0.19^**^	0.50	0.18	[0.14, 0.85]
Income	−0.02	−0.01	0.03	[−0.06, 0.05]
Desire perm position	0.22^**^	0.19	0.06	[0.06, 0.31]
Identification	−0.06	−0.01	0.02	[−0.04, 0.02]
Commitment	0.17	0.02	0.01	[−0.00, 0.05]
Perceived harm	0.26^***^	0.11	0.03	[0.04, 0.18]
Behavioral disengagement	0.06	0.03	0.04	[−0.05, 0.11]
Denial	−0.00	−0.00	0.06	[−0.13, 0.12]
Religious coping	0.25^***^	0.08	0.02	[0.03, 0.13]

Coding of sex: 1 = male and 2 = female.

** p < 0.01 two-tailed,

*** p < 0.001 two-tailed.

### Coping strategies among contingent faculty

Mean scores on each of the COPE variables, as presented in Table 3, are compared to mean scores as reported by Carver et al. (1989) in their sample of college students. Results of one sample *t*-tests reveal some statistically significant differences between the current sample and Carver et al.'s sample. Carver et al.'s sample report somewhat higher rates of religious coping (*t* = −8.47, *p* < 0.001, mean difference of 2.23), growth coping (*t* = −6.45, *p* < 0.001, difference of 1.28), denial (*t* = −12.29, *p* < 0.001, difference of 1.29), acceptance (*t* = −5.42, *p* < 0.001, difference of 1.11), mental disengagement (*t* = −4.54, *p* < 0.001, difference of 0.87), and suppression (*t* = −3.14, *p* < 0.01, difference of 0.57) than the current sample. Mean scores for humor and substance use are not available in Carver et al.'s sample. In summary, Carver et al.'s college student sample reported higher use of two engagement (growth, acceptance) and two disengagement mechanisms (denial, mental disengagement) than contingent faculty; there is no evidence that the two groups differ significantly in the ratio of use of engagement or disengagement mechanisms. The most notable difference between the two groups is that contingent faculty members in the current sample report a low rate of religious coping compared to the college student sample.

In the current sample, sex correlated with two coping mechanisms. Specifically, women reported significantly more use of seeking emotional support (*r* = 0.30, *p* < 0.001) and of planning (*r* = 0.22, *p* < 0.01) than did men. The result regarding support seeking is consistent with past research (Tamres et al., 2002). Age correlated with coping mechanisms such that older people utilized less mental disengagement (*r* = −0.22, *p* < 0.01) and substance use (*r* = −0.26, *p* < 0.001) than did younger people, indicating a tendency for older people to utilize less maladaptive coping mechanisms than younger people. These findings are generally consistent with past research (Aldwin, 2011).

### Contingent faculty experiences of depression, anxiety, and stress

Scores on the DASS variables in the current sample all fall within “normal” ranges for each of the three variables, as reported by Lovibond and Lovibond (1995).

Results in Table 6 revealed that depression correlated significantly and positively with the desire for a tenure-track position (*r* = 0.34, *p* < 0.01), commitment (*r* = 0.32, *p* < 0.01), perceived stressors (*r* = 0.34, *p* < 0.001), perceived harm (*r* = 0.38, *p* < 0.001), mental disengagement (*r* = 0.32, *p* < 0.01), venting (*r* = 0.28, *p* < 0.01), behavioral disengagement (*r* = 0.41, *p* < 0.001), substance use (*r* = 0.39, *p* < 0.001), and negatively with income (*r* = −0.38, *p* = 0.001). Anxiety correlated significantly and positively with commitment (*r* = 0.32, *p* < 0.01), identification (*r* = 0.35, *p* < 0.001), perceived stressors (*r* = 0.28, *p* < 0.01), perceived harm (*r* = 0.39, *p* < 0.001), and substance use (*r* = 0.44, *p* < 0.001) and negatively with income (*r* = −0.29, *p* < 0.01). Stress correlated significantly and positively with desire for a tenure-track position (*r* = 0.39 *p* < 0.001), commitment (*r* = 0.32, *p* < 0.01), perceived stressors (*r* = 0.30, *p* < 0.01), perceived harm (*r* = 0.39, *p* < 0.001), venting (*r* = 0.39, *p* < 0.001), behavioral disengagement (*r* = 0.32, *p* < 0.01), and substance use (*r* = 0.31, *p* < 0.01) and negatively with income (*r* = −0.30, *p* < 0.01). Desire for a tenure-track position, commitment, perceived harm, perceived stressors, and several disengagement coping mechanisms correlated positively with each of the three predicted variables (depression, anxiety, and stress), and income correlated negatively with all three variables. All predictor variables were measured at time 1 and the predicted variables (depression, anxiety, stress) were measured at time 2, 2–4 months later.

Regressions were conducted to determine which variables best predict depression, anxiety, and stress. Each regression equation included the following variables as predictors: all demographic variables, identification, commitment, harm, stress, and coping mechanisms for which Pearson correlations with the predicted variable were significant. In the regression model predicting depression, substance use was significant [β = 0.446, *p* < 0.001; *F*_(12, 62)_ = 4.29, *p* < 0.001; *R*^2^ = 0.51, adjusted *R*^2^ = 0.39]. In the regression model predicting anxiety, sex (β = 0.300, *p* = 0.006) and substance use (β = 0.367, *p* = 0.002) were significant [*F*_(9, 67)_ = 5.15, *p* < 0.001; *R*^2^ = 0.44, adjusted *R*^2^ = 0.36], with men reporting higher anxiety than women. When predicting stress, sex (β = −0.408, *p* = 0.001), age (β = −0.297, *p* = 0.01), and venting (β = 0.293, *p* = 0.008) were significant [*F*_(11, 63)_ = 4.65, *R*^2^ = 0.50, adjusted *R*^2^ = 0.39], with men reporting higher stress than women. Age was a negative predictor. Refer to Tables 9–11 for complete regression results. Pearson correlations between sex and anxiety, sex and stress, and age and stress were not significant while sex and/or age emerged as significant predictors in the regressions involving these variables. These discrepancies are due to missing data in the regressions; Pearson correlations involved sample sizes of about 90 whereas regressions involved sample sizes of about 65.

**Table 9:** **Standard multiple regression predicting depression**.

**Variable**	**β**	**Unstd B**	**Std Error**	**95% CI**
Age	−0.12	−0.08	07	[−0.23, 0.07]
Sex	−0.28	−5.43	2.19	[−9.83, −1.04]
Income	−0.07	−0.18	0.32	[−0.82, 0.45]
Desire perm position	0.13	0.80	0.76	[−0.72, 2.31]
Identification	0.09	0.11	0.17	[−0.22, 0.44]
Commitment	0.23	0.25	0.19	[−0.13, 0.64]
Perceived harm	−0.02	−0.07	0.49	[−1.04, 0.90]
Perceived stress	0.02	0.14	1.10	[−2.08, 2.36]
Behavioral disengagement	0.18	0.76	0.51	[−0.27, 1.78]
Mental disengagement	−0.02	−0.08	0.47	[−1.02, 0.86]
Venting	0.06	0.20	0.36	[−0.52, 0.93]
Substance use	0.45^***^	1.79	0.47	[0.85, 2.73]

Coding of sex: 1 = male and 2 = female.

*** p < 0.001 two-tailed.

**Table 10:** **Standard multiple regression predicting anxiety**.

**Variable**	**β**	**Unstd B**	**Std Error**	**95% CI**
Age	−0.13	−0.07	0.06	[−0.18, 0.04]
Sex	−0.30^**^	−4.53	1.60	[−7.74, −1.32]
Income	0.01	0.03	0.23	[−0.44, 0.49]
Desire perm position	0.05	0.23	0.55	[−0.88, 1.33]
Identification	0.28	0.27	0.12	[0.03, 0.51]
Commitment	0.16	0.13	0.12	[−0.11, 0.37]
Perceived harm	0.16	0.38	0.37	[−0.36, 1.12]
Perceived stress	0.06	0.36	0.82	[−1.28, 2.00]
Substance use	0.37^**^	1.18	0.36	[0.46, 1.90]

Coding of sex: 1 = male and 2 = female.

** p < 0.01 two-tailed.

**Table 11:** **Standard multiple regression predicting stress**.

**Variable**	**β**	**Unstd B**	**Std Error**	**95% CI**
Age	−0.30^**^	−0.21	0.78	[−0.37, −0.05]
Sex	−0.41^***^	−8.36	2.29	[−12.95, −3.77]
Income	0.01	0.04	0.32	[−0.61, 0.68]
Desire perm position	0.08	0.50	0.79	[−1.08, 2.07]
Identification	0.16	0.21	0.17	[−0.13, 0.55]
Commitment	0.02	0.02	0.19	[−0.36, 0.40]
Perceived harm	0.30	0.96	0.51	[−0.06, 1.97]
Perceived stress	0.08	0.61	1.16	[−1.72, 2.94]
Behavioral disengagement	−0.00	−0.01	0.52	[−1.05, 1.04]
Venting	0.29^**^	0.98	0.36	[0.26, 1.70]
Substance use	0.14	0.58	0.36	[−0.41, 1.56]

Coding of sex: 1 = male and 2 = female.

** p < 0.01 two-tailed,

*** p < 0.001 two-tailed.

### Organizational commitment among contingent faculty

The mean score for organizational commitment in the current study is comparable to results reported by Fuller et al. (2006), who studied organizational commitment in university employees (31 administrators, 131 staff, and 157 faculty) at a medium sized university in the United States utilizing Allen and Meyer's (1990) measure. Commitment in the current study (mean = 31.63) was higher than that reported by Fuller et al. (mean = 28.63, *t* = 4.17, *p* < 0.001). In our sample, organizational commitment did not correlate with age or sex, contrary to previous research which has shown higher levels of organizational commitment among older employees and women in non-academic employees (Mathieu and Zajac, 1990; McFarlin and Sweeney, 1992; Kacmar et al., 1999).

Six regressions were conducted to examine whether organizational commitment interacted with (a) perceived workplace harm and (b) perceived workplace stress in the prediction of depression, anxiety, and stress. None of the six regressions produced a significant interaction effect. Therefore, the six regressions failed to support the notion that organizational commitment buffered the effect of either perceived harm or perceived stress in the workplace on depression, anxiety, or stress.

## Discussion

Research on the impact of contingent faculty in higher education has tended to focus on the impact of contingency on universities and on students, with less attention paid to the experiences and psychological well-being of contingent faculty themselves. In particular, previous research has not identified common workplace stressors for contingent faculty nor has it addressed the impact of contingency on longer-term health outcomes such as depression and anxiety in the context of academic work. To address the latter, this study investigated predictors of perceptions of workplace stressors and harm and depression, anxiety, and stress among faculty who work off the tenure track. Specifically, we examined the role that demographic factors, situational variables (e.g., total family income), organizational commitment and identification, and individual coping mechanisms may play in shaping the psychological experiences of contingent faculty.

### Common workplace stressors and situational factors affecting stress, anxiety, and depression

A primary purpose of this study was to examine workplace stressors among NTT faculty. Research conducted in non-academic workplace settings finds that temporary work status is associated with stress, such that temporary workers report more stress than permanent employees (Benavides et al., 2000). We were not able to compare stressors or stress levels of NTT and tenure-track faculty in our study, but did identify common workplace stressors in our NTT sample. In response to an open-ended question about the most stressful aspects of their work, from which we obtained qualitative data, a substantial proportion of faculty in our sample listed contingency or the precariousness of their position as one of the most significant stressors that they experience. This finding is consistent with previous studies of workplace stress, which argue that temporary employment itself is a source of stress. NTT faculty in our sample also identified heavy workload, lack of institutional support such as access to a physical office, and low pay or pay inequity as significant sources of stress.

Much of our quantitative data corroborated the above results from our open-ended survey question. Two situational factors—lower family income and inability to find permanent work—emerged as risk factors across several different areas of our results. NTT faculty who would prefer a permanent position were more likely to perceive stressors in the workplace, and NTT faculty who would prefer a permanent position and those with lower family incomes were more likely to experience depression, anxiety, and stress. Together, these findings suggest that faculty who are financially insecure, and the estimated 50% of NTT faculty who desire full-time work may be particularly at risk for negative health outcomes. A desire for a permanent job may be an additional indicator of low or insufficient income—indeed, the two were correlated in our sample (*r* = −0.18, *p* < 0.05). Alternatively, a desire for a permanent job may represent an interest in the additional job security, resources, representation, status and recognition that a permanent position would bring. In the absence of permanent positions for all NTT faculty—some NTT positions will always exist since not all faculty desire permanent positions—providing as many of these benefits as possible may help to mitigate negative effects.

### Organizational commitment and identification

We also examined how NTT faculty's psychological attachment to their university, in the form of organizational identification and commitment, may impact their stress-related perceptions and experiences. We found that organizational commitment and organizational identification are associated with a tendency to see greater harm and to experience more stressors in the workplace. In particular, we found that organizational identification was weakly associated with perceptions of stressors and harm, whereas organizational commitment was moderately correlated with perceptions of stressors and harm. Identification represents a “first level” of commitment to a university that may or may not develop into a deeper dedication, in the form of organizational commitment. These findings suggest that the more connected NTT faculty are to an institution, the more likely they are to perceive stressors and harm. A psychological attachment to a university may make NTT faculty more attentive and sensitive to potentially harmful events occurring on campus.

Our results also indicated that organizational identification and commitment are associated with the extent to which faculty experience depression, anxiety, and stress. Faculty who were higher in organizational identification and commitment tended to report higher levels of anxiety. Additionally, faculty who were higher in organizational commitment (but not identification) tended to report higher levels of depression and stress. These findings suggest that a psychological attachment to an organizational may be a risk factor for temporary employees. The results of this study also suggest that, rather than acting as a buffer against stress as in previous research with other groups of employees (e.g., Meyer and Allen, 1997), organizational commitment may predispose NTT faculty to stress. Indeed, some recent research indicates that organizational commitment may be detrimental to temporary employees because it heightens the damage to well-being that occurs when temporary employees' work situations are modified outside their control (Galais and Moser, 2009). Our findings with regard to depression, anxiety, and stress are consistent with this possibility.

### Coping mechanisms

In general, NTT faculty in our sample used engagement and disengagement coping mechanisms at rates similar to those reported in a large undergraduate sample (Carver et al., 1989). Our correlational results support the notion that disengagement coping mechanisms such as denial, behavioral disengagement (giving up), and substance abuse, may predispose NTT faculty to perceiving harm and stressors in the workplace, and to depression, anxiety, and stress. In addition to calculating Pearson correlations between variables, we conducted regressions in order to determine which variables (demographic, situational, identification, commitment, and coping mechanisms) predicted perceived stressors, perceived harm, depression, anxiety, and stress, when all other variables were held constant. We found that, in the regressions which predicted depression and anxiety, one disengagement coping mechanism (substance use) emerged as a predictor, showing that substance use independently contributed to depression and anxiety, above and beyond the contribution of other variables, such as family income or inability to find a permanent job. In the regression which predicted stress, the coping mechanism venting emerged as an independent predictor. Venting, which involves letting out one's feelings with others, although not entirely “bad,” often appears to operate as more dysfunctional than functional, especially by comparison to more proactive coping mechanisms such as planning and active coping (Connor-Smith and Flachsbart, 2007). These results suggest that one intervention for NTT faculty could be teaching them to utilize functional coping mechanisms while discouraging the use of dysfunctional coping mechanisms.

### Demographic factors

With the expectation that individuals will experience and cope with stressors and stress in different ways, we were also interested in examining sources of variation in NTT faculty's experiences of stressors and stress. Consistent with previous research on contingent faculty experiences, our results indicated that demographic factors—age and sex—are associated with NTT faculty experiences of stressors and harm and may predispose one to depression, anxiety, and stress. In particular, older faculty were more likely to perceive harm in the workplace, and also reported lower levels of stress than their younger counterparts. Older faculty in our sample also tended to use less maladaptive coping methods than younger faculty. These findings are consistent with previous research on coping, which finds that older adults may use more efficient coping mechanisms and are better at regulating negative emotions, resulting in lower stress levels (Aldwin, 2011). However, older NTT faculty were equally likely to perceive stressors in the workplace, and reported similar levels of anxiety and depression, compared to younger faculty.

Consistent with previous studies, we found that among contingent faculty, women reported more social support seeking, and were more likely to perceive stressors at work. However, in the current study, sex was not correlated with depression, anxiety, or stress when Pearson correlations were calculated. In regressions involving anxiety and stress, which utilized a subset of the sample upon which Pearson correlations were calculated (due to missing data), men reported higher anxiety and stress than women. The findings regarding depression and anxiety are inconsistent with previous research utilizing the DASS and with knowledge about gender differences in clinical diagnosis. Specifically, in a large non-clinical sample of adults in a previous study, women scored significantly higher than men on both the Depression and the Anxiety scales of the DASS (Crawford and Henry, 2003). Additionally, diagnoses of major depressive disorder and of most anxiety disorders in the United States are more common among women than among men (American Psychiatric Association, 2013). Our findings may suggest greater psychological similarity between women and men NTT faculty than among women and men in the general population, at least in regard to experiences of depression and anxiety. Further research could investigate whether these findings are reliable.

Women and men in the current sample did not differ in regard to stress as measured by the Stress scale of the DASS, which is a measure of stress reactions. These findings are consistent with the results of Crawford and Henry's (2003) study utilizing the DASS Stress scale. Earlier research on gender differences in stress reactions has produced complex results. In general, women are more reactive to stressors than are men, for a wide variety of stressors, but if the stressors are work-related or financial, results have been inconsistent, with a slight tendency for research to show that men experience more extreme stress reactions to work-related and financial stressors than do women (Helgeson, 2011). Our own results mirror this inconsistency in the literature; as mentioned above, in our regression analyses conducted on a subset of our sample due to missing data, the pattern of results was somewhat different than the Pearson correlation results. In the regression analysis, women reported lower levels of stress, relative to their male counterparts.

### Future directions and limitations

Our research results show that contingent faculty who have lower family incomes, who have been unable to find a permanent position, who are most committed to their institution, and who use dysfunctional coping mechanisms are most likely to perceive higher levels of workplace stressors and harm and to experience higher levels of depression, anxiety, and stress. What can be done to help these faculty to achieve higher levels of well-being? The interventions that are most likely to be successful are those that occur at the institutional level, given that the institution controls access to resources which satisfy human needs at the most basic survival levels (e.g., income for all basic needs, job security to satisfy safety and security needs) and at higher levels of need (e.g., participation in governance to partially satisfy need for respect from TT peers). In our results, the relationship between a worker's commitment to their institution and their psychological well-being is the obverse of this relationship in most other samples of employees; committed employees, in other samples, typically experience fewer negative emotional states than those who are less committed. An employer or institution which fails to reward committed employees, and which instead behaves in ways that could be perceived by the employees as punishment (e.g., through classifying an employee as “temporary” for many years or decades, through failing to recognize the employee's contribution, through placing a low ceiling on pay) is doing a disservice not only to the employees but also to the institution itself. In the case of universities, harm to the faculty is likely to indirectly cause harm to the students and therefore to the mission of the institution. The reward for committed employees could, and should, include recognition, support, compensation, and participation in shared governance, which are factors that have been found to be associated with greater organizational commitment in employees in other samples (Wayne et al., 1997; Kacmar et al., 1999; Murphy, 2009).

Although interventions at the level of the institution are important, faculty may also be able to act individually by modifying their responses to workplace stressors. Two dysfunctional coping mechanisms (substance use and venting) contributed to one or more of the negative emotion outcomes variables (depression, anxiety, stress) independently of all other variables included in the regressions. Those NTT faculty who utilize dysfunctional coping mechanisms at a relatively high rate could commit to learning more functional coping mechanisms such as planning, active coping, and seeking instrumental (tangible) social support from others. Universities could offer coping workshops for employees. However, we caution that such training may prove more difficult than it may sound. It is possible that, among the NTT faculty in our sample, contingent work conditions may have led to or enhanced the development of the dysfunctional coping mechanisms. Possibly, for many or most of the individuals who are already suffering from depression, anxiety, or stress, changing oneself would do little to improve their well-being; a change in their circumstances may be required. Our uncertainty regarding the meaning of this finding reflects the primary limitation of our study, which is that it is correlational in nature and we cannot determine the causal relationships between many of our variables. Related to this, although we showed through our longitudinal design that a variety of variables (e.g., perception of workplaces stressors and harm, coping mechanisms) predicted depression, anxiety, and stress 2–4 months later, results which are consistent with the interpretation that workplace stressors, harm, and coping mechanisms partially cause levels of depression, anxiety, and stress, a stronger methodology is still needed to rule out alternative explanations of these correlational findings.

Future research should investigate the reliability of our findings that the most committed contingent faculty are also the ones whose well-being most suffers. We could extend this research by investigating the nature and deeper meaning of organizational commitment that occurs among some contingent faculty. Given that these faculty typically receive low pay and have little job security, and many have spent years in graduate school earning PhDs or other higher degrees, what types of sacrifices have they made in non-work areas of their lives? Do they have children, life partners, deep friendships? If their lives have involved sacrifice in these areas, what types of feelings do they have about having made these sacrifices? Is the amount of sacrifice in one's life outside of work associated with greater commitment to one's institution?

The study of psychological well-being among contingent faculty is uncharted territory. We have investigated relationships between several psychological variables, seeking to predict depression, anxiety, and stress in a group of contingent faculty. Hundreds of thousands of individuals in the United States work as contingent faculty, with millions of contingent faculty workers in the world (Curtis, 2014). Further research on factors which affect the well-being of this group of employees may lead to improvement in the lives of millions of contingent workers and their families.

### Conclusion

The present study examined experiences of stress and coping among NTT faculty. Due to a variety of trends in higher education, NTT faculty are a growing population whose contingent academic appointments are likely to produce unique stressors and possibly negative health effects. We found that NTT faculty perceive stressors at work that are related to their contingent positions. Demographic and situational factors, dysfunctional coping mechanisms, and organizational commitment and identification were associated with more negative psychological experiences. Our findings suggest possibilities for institutional intervention. Overall, we argue that universities would be well-served by attending to the needs of NTT faculty on campus in order to mitigate negative outcomes for institutions, students, and faculty. In tandem with improving the working conditions of NTT faculty, we support investigating the position that the AAUP (2014) takes—that the dramatic increase in NTT positions and decrease in tenure line positions that has occurred in recent decades may not be economically necessary.

### Conflict of interest statement

The authors declare that the research was conducted in the absence of any commercial or financial relationships that could be construed as a potential conflict of interest.
